# Effectiveness and Safety of the Combination of Paracetamol 1000 mg and Ibuprofen 300 mg Versus Ibuprofen 600 mg in Monotherapy in Acute Low Back Pain: Results from a Phase IV Randomized Study

**DOI:** 10.3390/jcm15052022

**Published:** 2026-03-06

**Authors:** Michal Harasymczuk, Antimo Moretti, Martina Barcaroli, Elisa Quarchioni, Ajona Tulipano, Adriano Nicolotti, Michela Procaccini, Enrica Salvatori, Agnese Cattaneo

**Affiliations:** 1 Traumatology, Orthopedics, Hand Surgery Department, Dega Orthopedics Institute, Poznan University of Medical Sciences, 60-812 Poznan, Poland; 2 Flosmed Medical Center, 60-192 Poznan, Poland; 3 Multidisciplinary Department of Medical and Surgical Specialties and Dentistry, University of Campania “Luigi Vanvitelli”, 80138 Naples, Italy; 4 Global Medical Department, Angelini Pharma S.p.A., 00181 Rome, Italy

**Keywords:** low back pain, ibuprofen in monotherapy, paracetamol and ibuprofen, combined therapy, effectiveness, safety

## Abstract

**Objectives**: This study aimed to evaluate the effectiveness and safety of paracetamol 1000 mg/ibuprofen 300 mg administered three times daily (TID) in comparison with ibuprofen 600 mg TID in the management of patients with acute moderate/severe non-specific low back pain (LBP). **Methods**: This was a phase IV, randomized, open-label, parallel-group study conducted in adults with moderate/severe LBP (Visual Analogue Scale [VAS] score ≥ 40 mm). **Results**: A total of 171 patients were included in the modified intention-to-treat (m-ITT) population (paracetamol 1000 mg/ibuprofen 300 mg: 83 patients; ibuprofen 600 mg: 88 patients). No significant between-group difference on the primary endpoint (SPID 0–3 days) was found. Patients were mainly women (60.2% and 55.7%), with a mean age of 42.8 and 43.3 years, respectively. In the m-ITT population, the effectiveness, safety and tolerability were similar between groups. In the per-protocol population, clinical pain reduction was observed with paracetamol 1000 mg/ibuprofen 300 mg. At visit 1, significant differences in the Clinical Global Impression–Improvement scale (paracetamol 1000 mg/ibuprofen 300 mg: 63.9%; ibuprofen 600 mg: 45.5%; *p* = 0.0137) and a trend favouring paracetamol 1000 mg/ibuprofen 300 mg in Patients’ Global Impression of Change (63.9% vs 44.4%; *p* = 0.0539) score were observed. **Conclusions**: Given the open-label design and the exploratory nature of study’s secondary endpoints, no claims of superiority can be drawn; but our findings confirm that good management of acute moderate/severe LBP can be achieved with multimodal therapy with paracetamol 1000 mg/ibuprofen 300 mg. EudraCT Number: 2020-005278-86 (EudraCT Number 2020-005278-86—Clinical trial results—EU Clinical Trials Register; date of registration: 14 June 2021).

## 1. Introduction

Low back pain (LBP) is defined as pain initiating from the region between the lower costal margin (lower edge of the ribs) and the inferior gluteal folds and is a leading contributor to disability worldwide [1]. It is more common among women and persons aged 40 to 80 years [2,3]. LBP limits physical activity, causing work absence [1], and is therefore one of the main reasons for work loss and causes a significant cost to society [2,4]. LBP may be classified by duration as acute (lasting <6 weeks), sub-acute (6–12 weeks), or chronic (>12 weeks) [5]. Acute LBP is often non-specific and therefore not attributable to a known cause [6], and often several physical (e.g., heavy/repetitive work or static postures) and psychosocial (e.g., stress, anxiety, depression) factors work in combination [7]. Diagnosis is generally based on self-reports of symptoms as well as medical history and physical examination [8].

Treatment for acute non-specific LBP should relieve pain, improve functional capacity, normalize muscle function and prevent recurrence and chronicity to allow patients to return to normal activities of daily living (ADL) as soon as possible, improving their quality-of-life (QoL) [7,9]. Analgesic drugs such as paracetamol, non-steroidal anti-inflammatory drugs (NSAIDs), and weak opioids are recommended in acute non-specific LBP management [7,8,10,11,12]. Some studies have investigated the use of these medications simultaneously to enhance the effect of the active ingredients [13,14,15,16,17] without increasing the dose [18,19,20,21]. In addition, clinical guidelines emphasize that patients with LBP should avoid bed rest and remain as active as possible, as maintaining movement supports recovery and reduces disability [22].

In this study a fixed-dose combination (FDC) medicinal product containing ibuprofen in association with paracetamol was used to obtain the greatest benefit of multimodal analgesia [23,24], since paracetamol exerts a central analgesic effect while ibuprofen exerts central and peripheral analgesic effects [25,26,27,28,29,30,31]. Moreover, a 600 mg dose of ibuprofen can offer stronger and sometimes longer pain control than a 300 mg dose; however, this also carries a higher risk of side effects [32]. Although the use of this combination is common in clinical practice [25,26,27,28,29], few recent clinical data are available on the short-term treatment of acute non-specific LBP [2]. This study aimed to evaluate the effectiveness and safety of the combination of paracetamol 1000 mg/ibuprofen 300 mg administered three times daily (TID), in comparison with ibuprofen 600 mg TID, in the management of patients with an acute non-specific LBP condition.

## 2. Materials and Methods

### 2.1. Study Population and Design

This was a phase IV, randomized, open-label, parallel-group, multicenter study (EudraCT: 2020-005278-86) conducted from 26 October 2021 to 5 October 2022 in Italy (3 sites) and Poland (9 sites). Adult patients aged between 18 and 64 years of age (limits included) with uncomplicated and localized acute LBP or acute exacerbation of chronic LBP, with moderate/severe pain at baseline (minimum Visual Analogue Scale [VAS] score ≥ 40 mm) were included in the study. Patients were excluded if they had known hypersensitivity or an allergy to the active ingredients and/or to any component of the study medications, any other NSAIDs or aspirin. Other exclusion criteria included: lactating and pregnant women, history of gastroduodenal ulcer or bleeding, history of severe cardiac, hepatic, or renal insufficiency, current anticoagulant therapy, previous treatment with anticoagulants in the seven days before the screening visit, concomitant use of physical or alternative therapies to treat current episode of pain, active or suspected esophageal, gastric, pyloric channel, or duodenal ulceration, or bleeding in the last 30 days, blood-formation disturbance, renal and/or hepatic failure, and acute hepatitis. Duration of patient participation in the trial (from informed consent form signature up to end of follow-up) was eight days (±1). Visit 1 was performed on day 4 (+1), right after the end of the treatment, and Visit 2/the final visit was performed on day 8 (+1), which is considered the end of the follow-up period.

The study was conducted in accordance with the Declaration of Helsinki Ethical Principles for Medical Research Involving Human Patients, applicable Good Clinical Practice principles and Good Pharmacoepidemiology Practice, and was approved by the Ethics Committees (Table S1). All patients signed the informed consent form. The study has been properly registered in the EudraCT database on 14 June 2021. Consolidated Standards of Reporting Trials (CONSORT) guidelines were followed for the preparation of this manuscript.

### 2.2. Experimental Protocol

The investigator assigned each patient the randomization number generated by the Clinical Supply Department of the sponsor. Patients were randomized 1:1 to receive 2 paracetamol 500 mg/ibuprofen 150 mg film-coated tablets TID for 3 days; or 1 ibuprofen 600 mg film-coated tablet TID for 3 days. A 3-day treatment period was considered adequate to control symptoms and minimize undesirable effects following the recommendations in the Summary of Product Characteristics (SmPC) [33,34]. Drugs were taken according to the SmPC (every 8 h ± 1 h) [33,34]. Since this was an open-label study, both the investigator and the patient were aware of the assigned medication.

### 2.3. Study Endpoints

The primary endpoint was the area under the pain intensity difference-versus-time curve of LBP scores up to 3 days of treatment (Sum of the Pain Intensity Differences [SPID] 0–3 days). The SPID was the sum of the average of 2 consecutive pain intensity differences from baseline multiplied by the time interval between the 2 time points.

The secondary endpoints of the study were: change from baseline (CFB) in VAS score up to the end of the study (visit 2) [35]; CFB in the hand-to-floor distance measured by a cm graduated bar; degree of improvement in functional disability, assessed by the Oswestry Disability Index (ODI) CFB; change in the patients’ global impression, measured by the Patients’ Global Impression of Change (PGIC) scale; change in the clinical global impression, measured by the Clinical Global Impression–Improvement (CGI-I) scale; the safety and tolerability.

The ODI contains 10 sections assessing how LBP affects the ability to manage ADL: pain intensity, personal care, lifting, walking, sitting, standing, sleeping, sexual life, social life, and travelling. Each section contains 6 statements scored from 0 (minimum degree of difficulty) to 5 (maximum degree of difficulty). The total score is obtained by summing up the scores of all sections, giving a maximum of 50 points. The final score is expressed as a percentage, meaning 0–20% suggests minimal disability; 21–40% suggests moderate disability; 41–60% suggests severe disability; 61–80% suggests very severe disability; and 81–100% suggests the individual is either bed-bound or exaggerating their symptoms [36].

The PGIC is a 7-point scale, from very much improved (1) to very much worse (7), with 4 meaning no change [37]. Patients gave information about their beliefs on the effectiveness of the treatment, evaluating all aspects of their health and QoL.

The CGI-I scale is a 7-point scale, from very much improved (1) to very much worse (7) since the initiation of treatment, with 4 meaning no change [38]. The investigator provided an overall clinician-determined summary evaluation of the treatment.

Safety and tolerability were assessed by monitoring the frequency of treatment-emergent AEs (TEAEs), adverse drug reactions (ADRs), and serious AEs (SAEs) in each treatment group. Medical histories and details of concurrent illnesses were collected at the screening visit. Vital sign measurements and laboratory analyses (hematology [including complete blood count], serum chemistry [including creatinine] and urinalysis) were performed at baseline, visit 1 and visit 2. The safety population (SP) was defined as all patients randomized to treatment and who received at least one dose of study medication.

### 2.4. Statistical Analysis

According to Merry et al. [13], considering the difference between the combination of ibuprofen and paracetamol and ibuprofen alone in the mean time-adjusted area under the curve at rest of 12.5 mm with a standard deviation of 20.2 mm, using a two-group *t*-test with a 5% two-sided significance level, a sample size of 69 patients per group is required with a power >90%. Assuming a drop-out rate of 20%, 176 patients (88 per treatment group) were planned to be enrolled. In addition, according to global guidelines, this population appears sufficient to detect average effects with 80% power [39].

Efficacy assessment was performed in the modified intention-to-treat (m-ITT) and per-protocol (PP) populations. The primary endpoint (SPID 0–3 days) was compared between groups using an ANCOVA model with treatment as a fixed effect and baseline VAS as a covariate; the model diagnostics were reviewed. The results are reported as adjusted mean differences with two-sided *p*-values. In addition, a standardized effect size (Cohen’s d) with 95% confidence intervals was computed for the primary endpoint. Analysis of covariance (ANCOVA) was applied to assess CFB to visit 1 and 2. Descriptive statistics were performed on the ODI, PGIC and CGI-I. A comparison between the rates of patients with AEs was performed by Chi-square test, Fisher’s exact test, or Cochran–Mantel–Haenszel test, as appropriate. Descriptive statistics were presented for the vital signs and physical examination. All analyses were performed with SAS^®^ version 9.4 (SAS Institute, Cary, NC, USA).

## 3. Results

### 3.1. Baseline Characteristics

**Figure 1:**
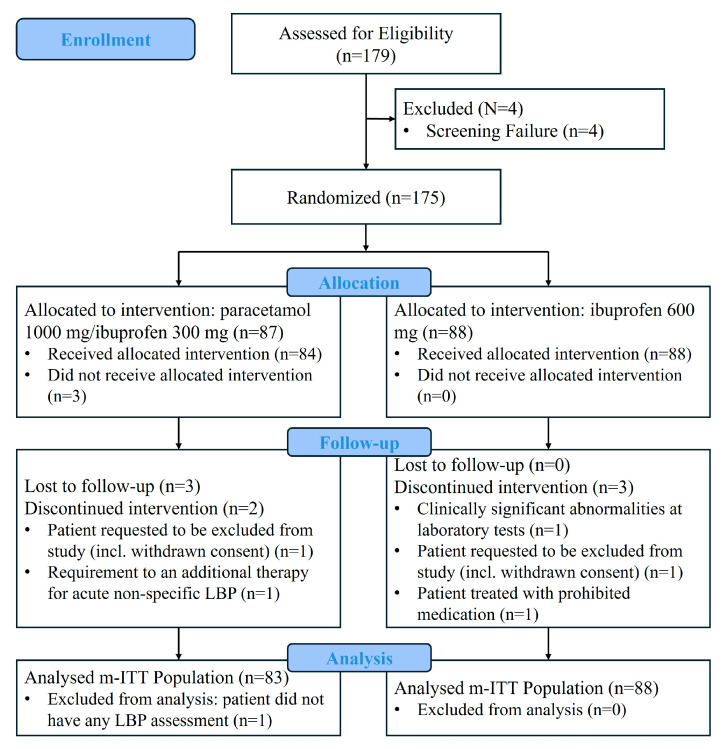
Consort diagram of study. m-ITT: modified intention-to-treat.

A total of 179 patients were assessed for eligibility. There were four screening failures, and 175 enrolled patients were randomized 1:1 to paracetamol 1000 mg/ibuprofen 300 mg (87 patients) and to ibuprofen 600 mg (88 patients). Three patients were excluded from the SP as they did not receive any dose of study medication. Therefore, the SP consisted of 172 patients: 84 in the paracetamol 1000 mg/ibuprofen 300 mg group and 88 in the ibuprofen 600 mg group. The m-ITT population consisted of 171 patients: 83 in the paracetamol 1000 mg/ibuprofen 300 mg group and 88 in the ibuprofen 600 mg group. Patients were considered evaluable in the PP analysis if the compliance was ≥80%. Nineteen patients were excluded from the PP population. Therefore, the PP population included 152 patients: 76 in the paracetamol 1000 mg/ibuprofen 300 mg group and 76 in the ibuprofen 600 mg group (Figure 1).

Eight patients discontinued the study before conclusion: five from the paracetamol 1000 mg/ibuprofen 300 mg group and three from the ibuprofen 600 mg group. The reasons for discontinuation were: lost to follow-up (three patients from the paracetamol 1000 mg/ibuprofen 300 mg group); consent withdrawal (one patient from the paracetamol 1000 mg/ibuprofen 300 mg group and one patient from the ibuprofen 600 mg group); clinically significant abnormalities at laboratory tests (one patient from the ibuprofen 600 mg group); prohibited medication use (one patient from the ibuprofen 600 mg group) and requirement of an additional therapy for acute non-specific LBP (one patient from the paracetamol 1000 mg/ibuprofen 300 mg group).

Overall, the demographic characteristics were similar in both groups (Table 1). In the m-ITT, 60.2% of patients included in the paracetamol 1000 mg/ibuprofen 300 mg group and 55.7% of patients included in the ibuprofen 600 mg group were women. The mean (standard deviation [SD]) age was 42.8 (11.1) and 43.3 (12.0) years, respectively.

### 3.2. LBP Assessment up to 3 Days

For the m-ITT population, the mean (SD) SPID values from baseline up to three days were −45.2 (33.0) and −41.0 (29.7) in the paracetamol 1000 mg/ibuprofen 300 mg and ibuprofen 600 mg groups, respectively (Table 2). No statistically significant differences in pain reduction were detected between groups. A clinical pain reduction was observed in the PP population for the paracetamol 1000 mg/ibuprofen 300 mg group in comparison with the ibuprofen 600 mg group (−46.2 [32.0] vs. −40.4 [30.0]; *p* = 0.0658).

The standardized effect size for the primary endpoint (SPID 0–3 days, m-ITT) was Cohen’s d = −0.134 (95% CI −0.434 to 0.166), indicating a small magnitude difference, consistent with the non-significant *p*-value.

### 3.3. Change in VAS Score from Baseline to Visit 2

LBP was reduced from baseline to visit 2 according to VAS score (Table 3). In the m-ITT population, the mean (SD) CFB of VAS score was −32.7 (21.3) and −32.2 (23.7) for the paracetamol 1000 mg/ibuprofen 300 mg and ibuprofen 600 mg groups, respectively, with no significant differences between groups. Similar results were observed for the PP population.

### 3.4. Change in the Hand-to-Floor Distance at Visit 2, Measured by a cm Graduated Bar

The mean (SD) CFB of the hand-to-floor distance was −4.3 (6.8) cm and −4.4 (6.2) cm for the paracetamol 1000 mg/ibuprofen 300 mg and ibuprofen 600 mg groups, respectively (Table 2), with no statistically significant differences between groups. Similar results were observed for the PP population.

### 3.5. Degree of Improvement in Functional Disability at Visits 1 and 2, Measured by the ODI

**Figure 2:**
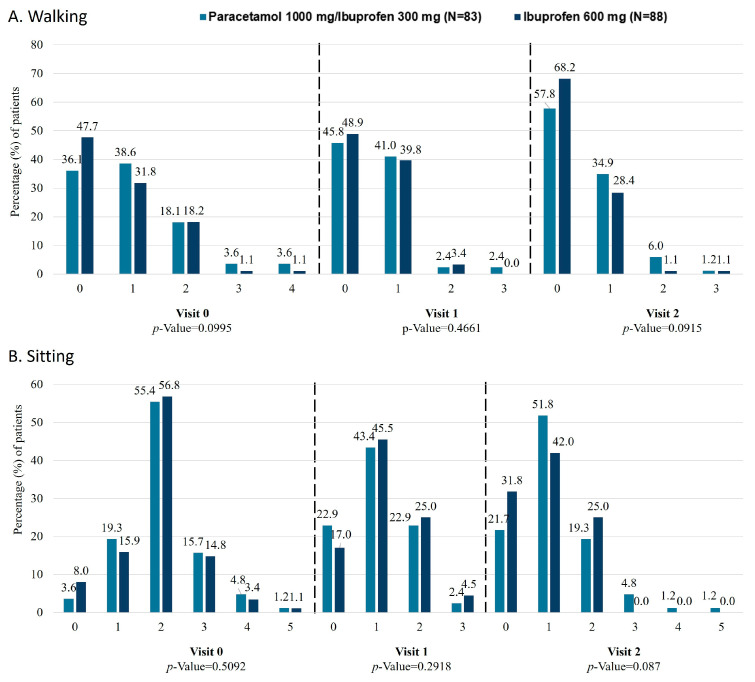
Degree of improvement in functional disability, measured by the ODI. 0 = I have no pain at the moment; 1 = the pain is very mild at the moment; 2 = the pain is moderate at the moment; 3 = the pain is fairly severe at the moment; 4 = the pain is very severe at the moment; 5 = the pain is the worst imaginable at the moment. ODI, Oswestry Disability Index.

According to the ODI, disability improved from baseline to visit 1 and visit 2 in 9 out of 10 sections, with the exception of sexual life. Although no significant differences were observed, trends towards a greater disability improvement for the ibuprofen 600 mg group in walking (*p* = 0.0915; Figure 2A) and for the paracetamol 1000 mg/ibuprofen 300 mg in sitting (*p* = 0.087; Figure 2B) at visit 2 were found. For walking, statistically significant differences in favour of the ibuprofen 600 mg group were also observed for the PP population (*p* = 0.0316).

### 3.6. Change in the Patients’ Global Impression at Visits 1 and 2, Measured by the PGIC Scale

**Figure 3:**
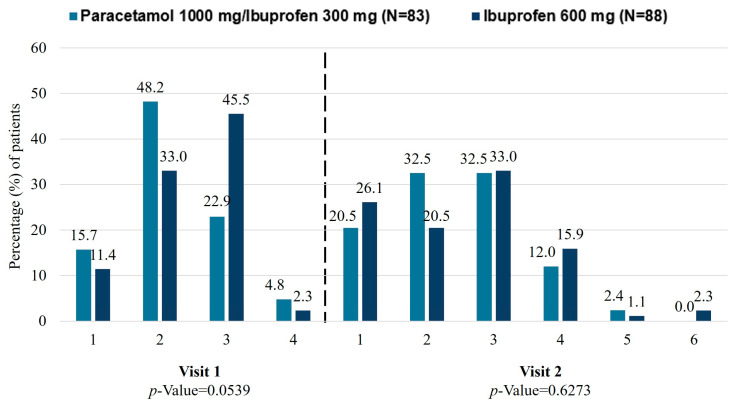
Change in the patients’ global impression, measured by the PGIC scale. 1 = very much improved; 2 = much improved; 3 = minimally improved; 4 = no change; 5 = minimally worse; 6 = much worse. PGIC, Patients’ Global Impression of Change.

In the m-ITT population, at visit 1, a total of 53 (63.9%) and 39 (44.4%) patients had improved LBP according to the PGIC scale (much improved/very much improved) for the paracetamol 1000 mg/ibuprofen 300 mg and ibuprofen 600 mg groups, respectively (Figure 3). This response was generally maintained at visit 2. Although no significant differences were observed, the analysis showed a trend towards a greater improvement in LBP for the paracetamol 1000 mg/ibuprofen 300 mg group (*p* = 0.0539) at visit 1. A statistically significant difference in improvement of LBP according to the PGIC scale was achieved with paracetamol 1000 mg/ibuprofen 300 mg compared to ibuprofen 600 mg at visit 1 (68.4% vs. 46.1%; *p* = 0.0341) in the PP population.

### 3.7. Change in the Clinical Global Impression at Visits 1 and 2, Measured by the CGI-I Scale

**Figure 4:**
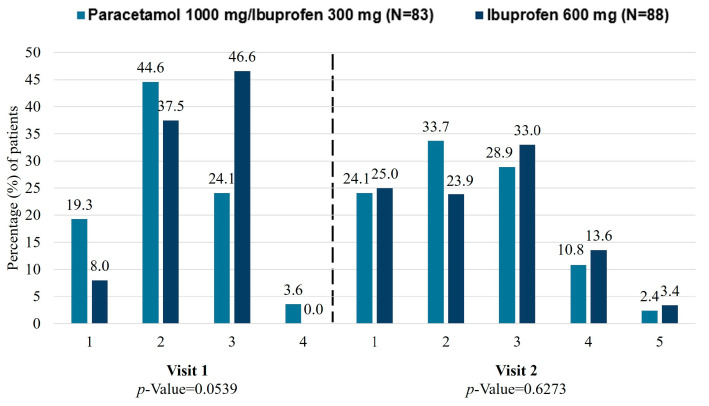
Change in the clinical global impression, measured by the CGI-I scale. 1 = very much improved; 2 = much improved; 3 = minimally improved; 4 = no change; 5 = minimally worse. CGI-I, Clinical Global Impression–Improvement.

At visit 1, a total of 53 (63.9%) and 40 (45.5%) patients achieved a CGI-I response (much improved/very much improved) for the paracetamol 1000 mg/ibuprofen 300 mg and ibuprofen 600 mg groups, respectively (Figure 4). The proportion of patients achieving a CGI-I response was higher at visit 2 compared to visit 1. At visit 2, 48 (57.8%) and 43 (48.9%) patients achieved CGI-I response (much improved/very much improved) for the paracetamol 1000 mg/ibuprofen 300 mg and ibuprofen 600 mg groups, respectively. The CGI was statistically significant (*p* = 0.0137) for patients receiving paracetamol 1000 mg/ibuprofen 300 mg in comparison with ibuprofen 600 mg at visit 1. Data for the PP population showed statistically significant differences between groups at visit 1 in favour of paracetamol 1000 mg/ibuprofen 300 mg (68.4% vs. 47.4%; *p* = 0.0097).

Overall, although a statistically significant difference was observed for the CGI-I scale at visit 1 and positive numerical trends were noted for PGIC and for the sitting item of the ODI, these findings were not consistent across endpoints or visits and did not persist in the primary efficacy assessment. Consistently with the non-significant between-group comparison on SPID 0–3 days, the standardized effect size (Cohen’s d) showed a small magnitude of difference, supporting the absence of a clinically meaningful advantage of either treatment over the 3-day window. As prespecified in the Statistical Analysis Plan, all of the secondary endpoints were exploratory/descriptive and were not subject to multiplicity adjustment; therefore, isolated differences should be interpreted with caution.

Within-group clinical relevance: Consistent with anchor- and distribution-based thresholds for acute pain, both treatment arms achieved clinically meaningful reductions in pain intensity from baseline over the 3-day window (mean ΔVAS m-ITT: −32.7 mm for paracetamol/ibuprofen and −32.2 mm for ibuprofen 600 mg), which exceed commonly cited MCID ranges for the VAS (≈15–30 mm depending on method and population) [40,41].

### 3.8. Safety

The mean (SD) exposure to the drug was 3.8 (0.55) days for paracetamol 1000 mg/ibuprofen 300 mg and 3.7 (0.49) days for ibuprofen 600 mg. Actual timing of dose administration (e.g., delayed or shifted doses) could extend the total exposure window. The most common concomitant conditions were hypertension (14.6% and 19.6%) and obesity (12.2% and 15.2%) in the paracetamol 1000 mg/ibuprofen 300 mg and ibuprofen 600 mg groups, respectively. A total of 37 patients in the paracetamol 1000 mg/ibuprofen 300 mg group and 36 patients in the ibuprofen 600 mg group received concomitant medication, the most common being vitamins (22.3% and 20.0%), sex hormones and modulators of the genital system (23.4% and 9.1%), and calcium channel blockers (12.8% and 14.5%).

A total of 63 TEAEs, mainly mild or moderate, were reported (Table 4). There were no serious TEAEs nor any leading to death. The most reported TEAEs in the paracetamol 1000 mg/ibuprofen 300 mg group were: medication error (three events; 11.1%), and overdose (three events, 11.1%). Whereas, in the ibuprofen 600 mg group, they were: headache (four events; 11.1%), upper abdominal pain (three events; 8.3%), diarrhea (three events; 8.3%), and nausea (three events; 8.3%). Gastrointestinal disorders were more common in the ibuprofen 600 mg group than in the paracetamol 1000 mg/ibuprofen 300 mg group.

No clear trend on any laboratory parameter change over time was observed. For platelets, a slight decrease in the ibuprofen 600 mg group, and slight increase in the paracetamol 1000 mg/ibuprofen 300 mg group was observed, whereas for triglycerides, an increase in mean value at visit 2 in the paracetamol 1000 mg/ibuprofen 300 mg group was found. The mean (SD) creatinine level was 0.8 (0.2) mg/dL at baseline and 0.8 (0.2) mg/dL at visit 2 (change from baseline: −0.0 [0.1]) in the paracetamol 1000 mg/ibuprofen 300 mg group and 1.8 (8.8) mg/dL at baseline and 1.9 (9.7) mg/dL at visit 2 (change from baseline: 0.1 [0.8]) in the ibuprofen 600 mg group. The mean (SD) aspartate aminotransferase level was 21.8 (13.8) U/L at baseline and 22.5 (11.3) U/L at visit 2 (change from baseline: 0.8 [13.9]) in the paracetamol 1000 mg/ibuprofen 300 mg group and 22.0 (7.1) U/L at baseline and 21.6 (7.1) U/L at visit 2 (change from baseline: −0.6 [5.2]) in the ibuprofen 600 mg group. The mean (SD) alanine aminotransferase level was 25.2 (24.8) U/L at baseline and 28.0 (18.6) U/L at visit 2 (change from baseline: 2.8 [17.4]) in the paracetamol 1000 mg/ibuprofen 300 mg group and 26.7 (16.0) U/L at baseline and 25.4 (12.7) U/L at visit 2 (change from baseline: −1.6 [8.0]) in the ibuprofen 600 mg group.

Considering the vital signs and physical examination, no statistically significant differences were observed from baseline to visit 2. Regarding the physical examination, in the paracetamol 1000 mg/ibuprofen 300 mg group, 37.5% of patients had varicose veins, 12.5% had lymphadenopathy, 12.5% had tonsillitis, 12.5% presented decreased mobility, 12.5% had hypokinesia and 12.5% telangiectasia. In the ibuprofen 600 mg group, 40.0% of patients presented decreased mobility, 20.0% were obese, 20.0% had rotator cuff syndrome and 20.0% had psoriasis.

## 4. Discussion

Combined use of paracetamol and ibuprofen is common in clinical practice [25,26,27,28,29]; however, few clinical data are available for the short-term treatment of acute non-specific LBP [2].

In this study, both paracetamol 1000 mg/ibuprofen 300 mg and ibuprofen 600 mg had comparable effectiveness for the relief of moderate/severe acute LBP. Although the mean SPID values and CFB in VAS score and in hand-to-floor distance showed a decrease over time, no statistically significant differences were observed between groups. Secondary endpoints were prespecified as exploratory/descriptive in the SAP, no multiplicity adjustment was planned or applied, and inferential claims were not drawn for the secondary analyses. A significant decrease (*p* = 0.045) in LBP intensity in patients treated with combined therapy for four days was observed in a randomized open-label study [2] comparing the efficacy and safety of ibuprofen 400 mg (40 patients) vs. ibuprofen 200 mg/paracetamol 325 mg (40 patients).

Moreover, statistically significant differences were obtained in the CGI-I at visit 1 and a positive trend was found in favour of paracetamol 1000 mg/ibuprofen 300 mg in the PGIC at visit 1 and for sitting disabilities in the ODI at visit 2. Although some patient- and clinician-reported scales showed numerical differences at visit 1, these were not supported by the primary endpoint, and the effect size confirmed that the between-group differences were not clinically meaningful. However, different studies [2,42,43], and a meta-analysis [44] in patients with LBP have found combination therapy to be more effective and faster than ibuprofen in monotherapy. Similarly, in postoperative endodontic [13,26,29,45] and acute postoperative [23] pain, the intensity of pain was reduced with the combination of paracetamol and ibuprofen in the immediate term. It would be interesting in future studies to investigate the effectiveness of paracetamol 1000 mg/ibuprofen 300 mg for LBP in the short term, during the first day of treatment. Furthermore, the combination of paracetamol and ibuprofen offers multimodal analgesia, since paracetamol exerts a central analgesic mode of action and ibuprofen central and peripheral modes of action, with the clinical advantage of a superior and longer-lasting analgesic effect compared to the single active ingredients taken as monotherapy [20].

Treatments were well-tolerated and had a good safety profile. Both ibuprofen and paracetamol can cause early toxicity due to mechanisms such as prostaglandin inhibition, the formation of reactive metabolites, or hypersensitivity, and the presence of individual factors (age, comorbidities, nutritional status, interactions) can accelerate or amplify these processes. Patients in the paracetamol 1000 mg/ibuprofen 300 mg group experienced fewer TEAEs than patients taking ibuprofen 600 mg (27 vs. 36 events), showing that paracetamol together with an NSAID did not increase the risk of AEs. As expected, in the ibuprofen 600 mg group, most of the TEAEs experienced during the study were of gastrointestinal nature, either of mild or moderate severity. Our results are in line with other studies on LBP [2,42,43], musculoskeletal pain [23,46,47], and in other diseases such as fever [48], upper and lower respiratory tract infections [49], or COVID-19 [50].

Since acute LBP is a condition with a high prevalence and incidence, our sample of 172 patients cannot be completely generalized to the whole population. Moreover, this was an open-label study, which could have influenced the results and interpretation, although treatment randomization was employed to minimize bias. Two visits (at day 4 and day 8 after treatment) could be too short a time to assess changes in the ODI, hand-to-floor distance, and global impression as measured by PGIC scale and could carry a high risk of random or placebo effects. In addition, comparing paracetamol 1000 mg/ibuprofen 300 mg with ibuprofen 600 mg results in different total daily doses of ibuprofen, which could complicate the interpretation of additive effects and disrupts group equivalence. Non-pharmacological therapies, physical activity, neuropathic screening (e.g. DN-4), detailed pain subtype classification and rescue medications were not considered and could substantially influence outcomes. Finally, our study was not designed to include a control place group nor a subgroup analysis (age, sex, body mass index, case of LBP, comorbidities). Despite these limitations, our findings add real-world evidence regarding the effectiveness and safety of the combination of paracetamol 1000 mg/ibuprofen 300 mg in treating LBP.

## 5. Conclusions

In this phase IV study, both paracetamol 1000 mg/ibuprofen 300 mg and ibuprofen 600 mg provided a clinically meaningful reduction in pain intensity over the 3-day treatment period, with improvements exceeding commonly reported MCID thresholds for acute and musculoskeletal pain (≈15–30 mm on the VAS). Consistent with the non-significant between-group difference on the primary endpoint and the small effect size, no clinically meaningful advantage of one regimen over the other was demonstrated. Overall, the fixed-dose combination represents a practical and well-tolerated multimodal analgesic option for the short-term management of acute non-specific low back pain and future blinded studies may further clarify potential early benefits.

## Figures and Tables

**Table 1:** Demographic and baseline characteristics of the m-ITT population.

	Paracetamol 1000 mg /Ibuprofen 300 mg	Ibuprofen 600 mg	*p*-Value
Age (years)			0.7729
*n*	83	88	
Mean (SD)	42.80 (11.05)	43.31 (12.03)	
Min–Max	19.00–64.00	21.00–65.00	
Gender			0.5474
Male	33 (39.8%)	39 (44.3%)	
Female	50 (60.2%)	49 (55.7%)	
Race			
White	83 (100.0%)	88 (100.0%)	

*n*, number; SD, standard deviation.

**Table 2:** LBP assessment (SPID) up to three days in the m-ITT population.

	Paracetamol 1000 mg /Ibuprofen 300 mg *n* = 83	Ibuprofen 600 mg *n* = 88	*p*-Value
SPID			0.1038
Mean (SD)	−45.2 (33.0)	−41.0 (29.7)	
Min–Max	−148.5–15.0	−142.0–25.0	

max, maximum; min, minimum; *n*, number; SD, standard deviation; SPID, Sum of the Pain Intensity Differences.

**Table 3:** Change in VAS score and the hand-to-floor distance from baseline to the end of study.

	Paracetamol 1000 mg/Ibuprofen 300 mg *n* = 83	Ibuprofen 600 mg *n* = 87 *	*p*-Value
VAS score			0.3132
Mean (SD)	−32.7 (21.3)	−32.2 (23.7)	
Min–Max	−69.0–20.0	−85.0–20.0	
HTFD			0.7610
Mean (SD)	−4.3 (6.8)	−4.4 (6.2)	
Min–Max	−39.5–8.3	−29.5–8.5	

HTFD, hand-to-floor distance; max, maximum; min, minimum; *n*, number; SD, standard deviation; VAS, Visual Analogue Scale. * In one patient, VAS scores and HTFD assessments were missing at visit 2.

**Table 4:** Overall summary of TEAEs.

	Paracetamol 1000 mg/Ibuprofen 300 mg	Ibuprofen 600 mg
Number of AEs, *n* (%)	27 (42.9)	36 (57.1)
Patients with AEs, *n* (%)	19 (22.6)	23 (26.1)
Number of ADRs, *n* (%)	13 (40.2)	19 (52.8)
Patients with ADRs, *n* (%)	11 (13.1)	14 (15.9)
Correlation of AEs with treatment, *n* (%)		
Certain	3 (11.1)	4 (11.1)
Conditional/Unclassifiable	1 (3.7)	4 (11.1)
Possible	6 (22.2)	9 (25.0)
Probable/Likely	4 (14.8)	6 (16.7)
Unlikely	13 (48.2)	10 (27.8)
Unassessable/Unclassifiable	0 (0.0)	3 (8.3)
Severity, *n* (%)		
Mild	17 (85.0)	25 (75.8)
Moderate	3 (15.0)	8 (24.2)
Outcome, *n* (%)		
Not recovered/Not resolved	3 (11.1)	3 (8.3)
Recovered/Resolved	16 (59.3)	29 (80.6)
Recovering/Resolving	1 (3.7)	1 (2.8)
Unknown	7 (25.9)	3 (8.3)
Action taken, *n* (%)		
Definitive interruption	1 (3.7)	4 (11.1)
No action because the patient is not under treatment	11 (40.7)	14 (33.9)
No change in therapy	15 (55.6)	18 (50.0)
Gastrointestinal disorders, *n* (%)		
Abdominal pain	1 (3.7)	2 (5.6)
Abdominal pain lower	0 (0.0)	1 (2.8)
Abdominal pain upper	1 (3.7)	3 (8.3)
Diarrhea	0 (0.0)	3 (8.3)
Dry mouth	1 (3.7)	0 (0.0)
Dyspepsia	0 (0.0)	1 (2.8)
Flatulence	0 (0.0)	1 (2.8)
Hemorrhoids	1 (3.7)	0 (0.0)
Nausea	0 (0.0)	3 (8.3)
Oesophageal discomfort	1 (3.7)	0 (0.0)
Nervous system disorders, *n* (%)		
Dizziness	1 (3.7)	0 (0.0)
Headache	0 (0.0)	4 (11.1)
Migraine	1 (3.7)	0 (0.0)
Somnolence	2 (7.4)	1 (2.8)
Injury, poisoning and procedural complications, *n* (%)		
Intentional product misuse	1 (3.7)	0 (0.0)
Medication error	3 (11.1)	2 (5.6)
Overdose	3 (11.1)	1 (2.8)

ADR, adverse drug reaction; AE, adverse event; *n*, number.

## Data Availability

The data used and/or analyzed during the current study are available from Angelini Pharma S.p.A upon reasonable request.

## Supplementary Materials

The following supporting information can be downloaded at https://www.mdpi.com/article/10.3390/jcm15052022/s1, Table S1: Ethics committees and date of approval.

## Abbreviations

The following abbreviations are used in this manuscript:

ADL	Activities of daily living
ADR	Adverse drug reaction
AE	Adverse event
CFB	Change from baseline
CGI-I	Clinical global impression–improvement
FDC	Fixed-dose combination
QoL	Quality-of-life
LBP	Low back pain
m-ITT	Modified intention-to-treat
NSAID	Non-steroidal anti-inflammatory drugs
ODI	Oswestry disability index
PGIC	Patient’s global impression of change
PP	Per protocol
SAE	Serious adverse event
SD	Standard deviation
SP	Safety population
SPID	Sum of the pain intensity differences
TEAE	Treatment-emergent adverse event
TID	Three times daily
VAS	Visual analogue scale
