# Supplementary material for: Effectiveness and Safety of the Combination of Paracetamol 1000 mg and Ibuprofen 300 mg Versus Ibuprofen 600 mg in Monotherapy in Acute Low Back Pain: Results from a Phase IV Randomized Study
Source: J Clin Med. 2026 Mar 6;15(5):2022. doi: 10.3390/jcm15052022 (PMC12985560; doi:10.3390/jcm15052022)
Supplement: Supplementary file 1 [file jcm-15-02022-s001.zip › jcm-4135122-supplementary/jcm-4135122-supplementary.pdf]

Supplementary Material

Table S1. Ethics Committees and date of approval

Center Name	Ethics Committee	Date of Approval
Azienda Ospedaliero Universitaria Policlinico “G.Rodolico - San Marco” U.O. Clinica Ortopedica	Comitato Etico Catania 1	19Apr2021
Azienda Ospedaliero Universitaria dell’Università degli studi della Campania Luigi Vanvitelli	Comitato Etico Università degli Studi della Campania “Luigi Vanvitelli” – A.O.U. “Luigi Vanvitelli”, A.O.R.N. “Ospedali dei Colli”	27Jul2021
Azienda Ospedaliera Universitaria Integrata Verona	Comitato Etico per la Sperimentazione Clinica (CESC) delle Province di Verona e Rovigo	07Jul2021
Centrum Medyczne Pratia Poznań	Komisja Bioetyczna przy Okręgowej Radzie Lekarskiej Wielkopolskiej Izby Lekarskiej	21Apr2021
Zespół Porani Specjalistycznych Reumed Wallenroda Filia nr 1	Komisja Bioetyczna przy Okręgowej Izbie Lekarskiej w Lublinie	21Apr2021
Centrum Medyczne Reuma Park	Komisja Bioetyczna Okręgowej Izby Lekarskiej w Warszawie	21Apr2021
Nasz Lekarz Przychodnie Medyczne (renamed to MICS Centrum Medyczne Torun)	Kujawsko-Pomorska Okręgowa Izba Lekarska w Toruniu	21Apr2021 (15Jun2022)
IN-VIVO Bydgoszcz	Komisja Bioetyczna przy Bydgoskiej Izbie Lekarskiej	21Apr2021
Przychodnia "Przy Szpitalu"	Komisja Bioetyczna przy Bydgoskiej Izbie Lekarskiej	21Apr2021
Centrum Medyczne Pratia Warszawa	Komisja Bioetyczna Okręgowej Izby Lekarskiej w Warszawie	21Apr2021
Centrum Medyczne AstiMed	Komisja Bioetyczna Okręgowej Izby Lekarskiej w Warszawie	21Apr2021
